# Calculating Ensemble Averaged Descriptions of Protein Rigidity without Sampling

**DOI:** 10.1371/journal.pone.0029176

**Published:** 2012-02-22

**Authors:** Luis C. González, Hui Wang, Dennis R. Livesay, Donald J. Jacobs

**Affiliations:** 1 Department of Bioinformatics and Genomics, University of North Carolina at Charlotte, Charlotte, North Carolina, United States of America; 2 Department of Physics and Optical Science, University of North Carolina at Charlotte, Charlotte, North Carolina, United States of America; Université d'Evry val d'Essonne, France

## Abstract

Previous works have demonstrated that protein rigidity is related to thermodynamic stability, especially under conditions that favor formation of native structure. Mechanical network rigidity properties of a single conformation are efficiently calculated using the integer body-bar Pebble Game (PG) algorithm. However, thermodynamic properties require averaging over many samples from the ensemble of accessible conformations to accurately account for fluctuations in network topology. We have developed a mean field Virtual Pebble Game (VPG) that represents the ensemble of networks by a single effective network. That is, all possible number of distance constraints (or bars) that can form between a pair of rigid bodies is replaced by the average number. The resulting effective network is viewed as having weighted edges, where the weight of an edge quantifies its capacity to absorb degrees of freedom. The VPG is interpreted as a flow problem on this effective network, which eliminates the need to sample. Across a nonredundant dataset of 272 protein structures, we apply the VPG to proteins for the first time. Our results show numerically and visually that the rigidity characterizations of the VPG accurately reflect the ensemble averaged 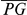 properties. This result positions the VPG as an efficient alternative to understand the mechanical role that chemical interactions play in maintaining protein stability.

## Introduction

The set of accessible conformations of a protein is critically dependent upon the arrangement and strength of chemical interactions, which greatly influences the conformational entropy. While there are many different computational models available to characterize protein dynamics [1]–[4], the computational efficiency and relative accuracy have made network rigidity models particularly attractive [5]. Describing protein structure as a network of constraints that fix the distance between atoms (vertices), the salient feature of network rigidity is to carefully characterize the number of degrees of freedom (DOF) within the network. The number of accessible DOF is *generally* reduced as chemical interactions are added to the network, which is related to the reduction in phase space upon formation of the interaction. In particular, adding a distance constraint to a flexible region reduces the number of available DOF, while adding one to an already rigid region does not.

While there are a number of graph theoretic algorithms to calculate network rigidity [6]–[9], the efficient Pebble Game (PG) has emerged as the most popular way to account for protein flexibility [5]. Indeed, various methods based on PG have been developed to analyze network rigidity [10]–[12], where FIRST [5] has served as the starting point for methods that explore the native conformational dynamics, such as ROCK [13], [14] and FRODA [15], [16]. Using pebbles to refer to DOF, network rigidity properties of the complete network are quickly calculated based on a strict accountancy of pebbles. Once complete, the PG identifies all flexible/rigid regions within the network. Unfortunately, FIRST, ROCK and FRODA are limited by an athermal formulation, meaning fluctuations within the noncovalent interaction network are not modeled.

Within molecular networks [17], covalent bonds are modeled as quenched constraints (meaning they are ever-present), whereas noncovalent bonds fluctuate on and off. The intermittent nature of the noncovalent interactions reflecting protein dynamics further complicates calculation of average network properties. In this direction, we have developed a statistical mechanical Distance Constraint Model (DCM) [18], [19] that is based on a Gibbs ensemble of PG networks that uses network rigidity to account for enthalpy-entropy compensation [20], [21]. The result of the DCM approach is that the give and take between protein stability and flexibility is accurately quantified [22]–[27]. In all works to date, solving of the DCM for protein structures has required average network rigidity properties determined from Monte Carlo sampling across a large sample of network topologies. In this approach, there is a binary on/off designation based on the probability of a constraint to be present or not. This randomness leads to an astronomically large ensemble of networks consisting of 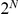 possibilities for 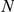 constraints that are fluctuating on or off throughout the network. Typically 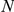 will range between a few hundred to several thousand in applications. Monte Carlo sampling works markedly well because of self averaging properties of constraint networks. It has been found that for statistical error bars to be within acceptable limits, millions of networks are usually necessary to be sampled [19].

Because there can be more than one distance constraint placed between a pair of rigid bodies, the body-bar PG [28] represents the network as a multi-graph, where more than one edge can connect between a pair of vertices. That is, each vertex represents a rigid body, having 6 DOF, and each edge represents a distance constraint. Herein, the framework of the body-bar PG algorithm is generalized, and, interestingly, requires only a minor modification in a way that preserves essentially the same implementation. That is, we have developed a Virtual Pebble Game (VPG) that allows for probabilistic descriptions of the network. The network now has only one edge between a pair of vertices with an assigned weight that defines the capacity for it to absorb DOF. This capacity is given by the average number of constraints that can form between a pair of vertices, thus it needs not be an integer value. The VPG extends the counting of constraints and DOF to real numbers, allowing for fractional DOF, which are viewed as representing the probability to find a DOF. Through this generalization of the PG implementation, the VPG algorithm tracks probability flow that governs where the average number of DOF pool within the effective network, rather than track individual DOF that fluctuate about this average. This approach leads to a dramatic computational speed-up because the PG algorithm dictates sampling over many networks to calculate equilibrium properties, whereas the VPG can probabilistically determine them from a single calculation. The approach of the VPG to calculate ensemble properties without sampling is in the same spirit as other algorithms that tackle important computational biology problems with a very large search space that otherwise would require excessive computation time [29]–[32].

In a recent report [33], we have demonstrated that the VPG closely reproduces the ensemble averaged counting of DOF within a variety of disordered lattices. The ensemble averaged results over many PG runs is designated 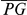. In this report, key average or consensus network rigidity metrics are directly compared across a non-redundant data set of 272 protein structures. For example, identified rigid clusters represent groups of vertices that behave as a single body. Numerically and visually we show that the VPG rigidity calculations faithfully represent an overwhelming majority of the ones performed by the PG. Varying the number of interactions present in the network allows us to identify the rigidifying effect that they have on protein structure [11]. Through a continuous increase in number of hydrogen bond (H-bond) constraints placed in the protein, a rigidity percolation is defined where the network progressively becomes more rigid. The rigidity threshold [34] defines the point where the protein just transitions from being globally flexible to globally rigid, or vice versa. At this rigidity threshold, the greatest fluctuation in network topology occurs, leading to the greatest differences between the 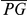 and VPG quantities. Remarkably, at the rigidity threshold, the similarity in all network rigidity metrics that were calculated using 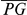 and VPG is found to be quantitatively high. As we demonstrate below, the VPG is ideally positioned as a viable alternative to ensemble averaging in the characterization of protein rigidity.

## Materials and Methods

### Protein Structure Description

We consider a dataset composed of 272 protein structures that are nonredundant at the SCOP [35] family level. Our dataset includes one, two and three domain proteins for PDB codes (see Table 1), that range from 50 to 764 residues. We focus on three types of chemical interactions, which are: *intra-residue, linker and hydrogen bond*. Note that salt-bridges are considered a special type of H-bond as described previously [5]. The intra-residue interaction models the covalent bonds that exist within a residue. The linker interaction represents the peptide bond that connects the C-N terminal atoms in adjacent residues. The reason we make a distinction between these two types of covalent bonds is due to the number of DOF they consume. While an intra-residue covalent bond (and disulfide bonds if any) consumes five DOF (leaving one for the dihedral angle), the linker consumes six DOF (locking any possible rotation) due to the partial double bond character of the amide group. The last interaction is the H-bond, which we specifically control whether a H-bond is present or not by the parameter 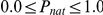. In this fashion, all possible H-bonds within the structure are present when 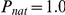, whereas no cross-linking H-bonds exist when 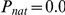. An independent H-bond consumes three DOF in order to account for the distance and angular constraints it imposes.

**Table 1:** PDB codes of the proteins in the dataset.

12AS	1A1X	1A32	1A3A	1A76	1A8L	1A92	1A9N	1AEP	1AF7	1AHO	1AHS	1AIH	1AK0	1AKO	1AL3	1ALV
1ALY	1AM9	1AN9	1AOC	1AOL	1ASH	1ATZ	1AVQ	1AYO	1B1C	1B3A	1B3T	1B5P	1B67	1B77	1B9O	1BAZ
1BBH	1BEA	1BF6	1BGV	1BIF	1BJA	1BKR	1BM8	1BOL	1BRT	1BTN	1BUP	1BX4	1BXY	1BYK	1C1D	1C3G
1C3P	1C4Q	1C5E	1C7K	1C7Q	1C8U	1CC5	1CCZ	1CHD	1CI6	1COJ	1COL	1CQ3	1CQY	1CSH	1CTF	1CV8
1CY5	1CYX	1D4T	1D7P	1D9C	1DFU	1DGW	1DJ7	1DK0	1DK8	1DKQ	1DL5	1DQ3	1DQG	1DQP	1DRW	1DSZ
1DTD	1DZF	1E2W	1E44	1E5K	1ECS	1ED1	1EE6	1EEJ	1EEM	1EFD	1EFV	1EGW	1EJE	1EKG	1EL6	1ELK
1EM8	1EP3	1EQF	1EWF	1EZ3	1F02	1F08	1F0K	1F20	1F5V	1F60	1FD9	1FN9	1G6S	1G73	1G8E	1GAK
1GL4	1GP0	1GQV	1GS5	1GWU	1GWY	1GXJ	1GYX	1H03	1H2C	1H2S	1H8P	1HW1	1HXN	1I0V	1I2A	1I2K
1I3J	1I4M	1I6P	1I78	1I8N	1IIB	1IO1	1IQ4	1IS3	1ISU	1J2L	1J2Z	1J71	1JDC	1JFL	1JH6	1JIW
1JKE	1JOV	1JSD	1JTD	1JUV	1JYH	1K6K	1KEA	1KID	1KNW	1KPT	1KQ3	1KTH	1L5O	1LAM	1LBV	1LGH
1LJ5	1LJO	1LKO	1LLM	1LMB	1LP1	1LYV	1M2K	1M9Z	1MDL	1MLA	1MML	1MSC	1MW7	1N69	1N81	1NH1
1NKD	1NPE	1NRZ	1NTY	1NYK	1O9Y	1OA8	1OAI	1OGD	1OK0	1OKC	1ON2	1OQV	1ORS	1OYG	1P1M	1P6O
1PDO	1PF5	1PTQ	1PUC	1PVM	1PYO	1QB2	1QEX	1QYN	1R7L	1RMD	1RP0	1RQW	1S12	1SCZ	1SFP	1SIQ
1SKN	1SQU	1SR8	1SVB	1SYX	1T5J	1T8K	1T9I	1TFE	1TKE	1TO6	1TUA	1TZV	1U0M	1UHE	1UUN	1V71
1V77	1VMO	1VP2	1VYI	1VZI	1VZY	1WQJ	1YU0	1ZDY	1ZJC	2AG4	2AVU	2B9D	2BH1	2CFQ	2CLY	2D5B
2EDM	2FCW	2G64	2I06	2IZY	2O39	2O4T	2OEB	2P62	2PHC	2PSP	2QFA	2RFT	2SIC	2UUI	2VO9	3COQ

**Figure 1:**
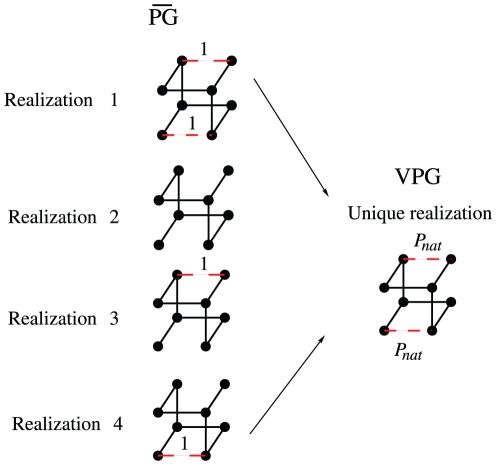
The respective network descriptions are compared. Equilibrium rigidity properties (designated as 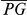) are calculated by averaging across an ensemble of binary networks where H-bonds are either present or not. Conversely, the VPG describes the network with H-bond probabilities.

A constraint topology file (CTF) contains a list of all the possible interactions that are to be considered within a specific protein structure. It is constructed from the original PDB file. The CTF defines each interaction type, as well as their probabilities. Quenched covalent interactions never change from one CTF to another, whereas the probability for a H-bond to form is described by the variable 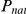. Fig. 1 compares the PG and VPG descriptions of a toy network with eight nodes, where quenched covalent bonds are solid and H-bonds are dashed. Two possible H-bonds exist in this example, leading to an ensemble of 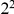 PG networks. Within each realization, the H-bond is either fully present with probability 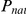 or not with probability 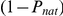. The 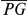 properties are determined by averaging over the ensemble generated by Monte Carlo sampling. Conversely, the VPG requires only one probabilistic network to describe the ensemble because the presence of a H-bond is directly quantified by its probability, 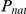, to be present.

### The VPG Algorithm

The three main elements of the PG algorithm are *pebbles*, *vertices* and *distance constraints*, which respectively represent DOF, rigid bodies and intramolecular interactions [28]. Note that the justification for the mapping between atoms and rigid bodies, and switching the PG applicable on a bar-joint network to a body-bar network are thoroughly explained in prior works [10], [28]. When the body-bar pebble game initiates, all vertices are unconnected and each is ascribed six free pebbles that describe its position and orientation in 3D. When an *independent* interaction is placed into the network, six trivial DOF are fixed on either one of its incident vertices while the number of distance constraints modeling it are consumed. In the language of the PG algorithm, distance constraints are recursively added to the network, and free pebbles cover the new constraints if they are independent, accounting for DOF removal from the system. Pebbles are not always locally available, but can often be transfered from remote regions of the network. That is, network rigidity is a long-range interaction that can propagate across the network [36], [37]. This pebble search function is possible given that pebbles provide directionality in the network, dependent upon which vertex has provided them. A constraint is *redundant* when a pebble cannot be transferred to cover it.

The search for pebbles in the directed network resembles a network flow problem [38], [39], given that the covered capacity of any edge will determine the maximal flow of pebbles through that edge. In a recursive fashion one edge at a time is placed in the network, always following the described process. This way the PG accomplishes its main goal, determining if an edge is independent (fully covered) or redundant (partially or none covered). When a search for pebbles fails and consequently a redundant constraint is found, all the vertices that were involved in the search collapse into a single vertex (with its six trivial DOF), and defines a minimally rigid graph [40], which we loosely refer to as a Laman subgraph [41] because of the analogous concept in two dimensions. This fact allows the PG to run virtually in linear time with the number of vertices in protein-like networks.

The crucial difference between the VPG and the original PG algorithm is the assignment of pebble capacity to edges, and to handle fractional pebbles. The capacity of an interaction represents the maximum number of DOF that it can consume; for linker and intra-residue interactions the capacity is six and five DOF, respectively. The capacity of a H-bond is defined as the product of its probability, 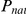, and the number of distance constraints used to model it, which is three. Therefore, consider a network consisting of vertices 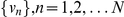, with a list of edges 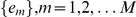. The capacity for the 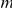-th edge is denoted by 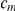. The VPG follows the following procedures and operations:

1. Initialize the graph with a set of isolated vertices 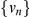, with the free DOF of each vertex 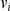 being 6.
2. From the list of edges 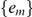, insert edge 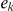 with capacity 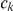 into the graph. Let 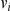 and 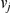 be the two incident vertices for edge 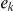.
3. Collect 6 pebbles for vertex 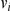 by doing a breadth first search.
4. Flag vertex 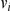 as visited, try to collect 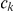 pebbles for vertex 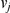 by doing a breadth first search while holding the 6 pebbles on 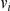 in place. If not all 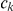 pebbles can be found in one trial, continue to collect more pebbles by carrying out the search repetitively until there are enough free pebbles on 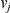 to cover edge 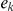, or if no new pebbles are found (a failed search).
5. If 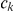 or more pebbles are collected on vertex 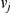, cover edge 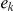 with 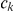 pebbles. Otherwise, all the visited vertices within the failed search are condensed into a single vertex. If 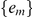 is not empty, go to step 2.
6. End of VPG.

### Rigid Cluster Decomposition

After having placed all the constraints, the PG and VPG algorithms determine the number of DOF left in the network. The trivial case is when there are just six DOF remaining, indicating that the network is globally rigid and all vertices are contained in a single rigid cluster. When there are greater than six remaining DOF, pebble location identifies which regions of the protein network are flexible or rigid. Excess pebbles identify flexible regions, whereas rigid regions occur when no free pebbles beyond 6 are accessible. From this information, it is possible to apply a *Rigid Cluster Decomposition* (RCD) to localize groups of vertices that move together as a rigid body. A rigid cluster is a subgraph with all of its vertices completely rigid among themselves.

**Figure 2:**
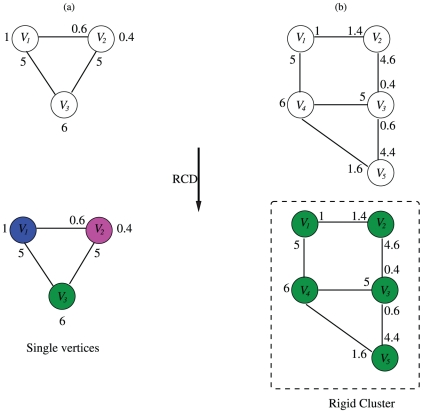
Two different rigid cluster decomposition examples are compared. In the first example, (a), there are 1.4 free pebbles available (located on vertices 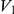 and 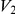), whereas the capacities of edges 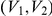, 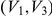, and 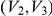 is, respectively, 0.6, 5.0, 5.0. If a hypothetical edge is added between any pair of vertices, there is always going to be possible to find DOF, therefore the three vertices result in single bodies (highlighted by color differences). Conversely, in the second example, (b), only the six trivial DOF can be found (on vertex 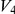). That is, no free pebbles remain in the network (they have all be consumed by the edges). As such, the five vertices belong to a single rigid cluster.

The process of finding rigid clusters in the VPG proceeds as follows: for any pair of vertices add a hypothetical edge, then try to cover it with 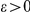 DOF, while six DOF are fixed on one of the incident vertices. If an excess number of DOF is found, then both vertices do not belong to the same rigid cluster, otherwise a failed search is declared and all the vertices involved in the search are part of the rigid cluster. Fig. 2 presents two example RCD cases. Notice that all the edges have been covered and they have different capacities. In the first case (Fig. 2a), there is a total of 7.4 available DOF. Therefore for any pair of vertices in the network, it will always be possible to gather 6+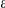 DOF with 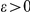 representing excess DOF, which indicates all constraints are independent and the network is globally flexible. For the second case (Fig. 2b), the number of available DOF is exactly six (on vertex four). Therefore, no excess DOF (i.e. 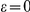) will be found under any circumstance, and this condition indicates that a rigid cluster is present that includes all five vertices.

It is worth emphasizing that the point of these examples is to show that the data structure for the VPG is essentially the same as the PG. On the other hand, the edge pebble capacities are not shown in Fig. 2 for either example. Yet, it can be surmised that the capacities of each edge in example (a) is precisely equal to the numbers assigned to the edges (i.e. 5, 5 and 0.6). If this were not the case, assuming one of these edges had a greater capacity, then some or all of the excess 1.4 DOF that is currently remaining would be used to cover the edges. Conversely, because there are no excess DOF in example (b) it is clear that either all the edges are being covered at their maximum capacity, or, their capacity is larger than the sum of the number of pebbles that cover the edge (on both sides must be added). If the capacity of an edge is larger than the total amount of pebbles covering it, then this would indicate that the edge is redundant. If this were the case, many vertices could be collapsed into a single vertex as explained above in regards to failed pebble searches, and creation of Laman subgraphs.

### Network Similarity Metrics

We employ two distinct metrics to compare the networks identified by the VPG and PG algorithms. To quantify rigid cluster similarity, we employ the Rand Measure (RM) [42]. The RM is a very well suited metric to compare clustering within a network. In the case of rigid cluster decomposition, both the body-bar PG and its VPG counterpart assign a unique label to each vertex to indicate the cluster it belongs to within the network. The network will generally consist of many rigid clusters. The RM is a combinatory count of all possible pairs of vertices where it counts all the cluster composition coincidences between the two networks generated by the two approaches. If both networks have the exact same rigid cluster decomposition, then RM is equal to 1. In general the RM has a range between 0 to 1. Zero is only possible if one network consist of all vertices within one cluster, while in the other network all vertices are in separate clusters (each vertex has its own unique label).

For a specific pair of vertices, there are two cases in which a match is found between the two networks. In the first case, the two vertices in network 1 belong to the same cluster (they have the same cluster label) and likewise, in network 2 both vertices belong to the same cluster. In the second case, the two vertices have different labels in network 1, indicating they belong to two different clusters, and likewise, in network 2 the two vertices belong to two different clusters. On the other hand, a match is not found if in one network the two vertices belong to the same cluster, while in the other network they belong to two different clusters. The RM is calculated by the total number of matches divided by the total number of possible pairs. A RM greater than 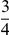 is a strong indicator of good agreement. A formal definition as given in [42] is: given 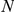 points, 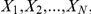 and two clusterings of them 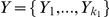 and 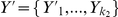 there is defined
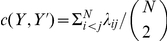
where 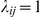 if 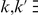 such that 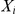 and 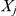 are in both 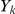 and 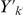 or if 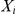 is in both 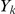 and 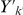 while 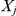 is in neither 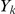 nor 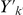, otherwise 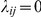.

We also compare the rigidity assessment between the majority vote from PG to the VPG assignment for each non-linker torsion bond in a protein. This provides a very sensitive metric to assess how well the VPG reflects the consensus results from a large sampling of PG runs. That is, we count the number of times that both approaches agree in their rigid versus flexible assessment, normalized by the total number of comparisons. This calculation leads to an agreement measure (AM) that ranges from −1 to 1. When the rigidity estimate from VPG matches the majority vote among all PG realization (i.e., a rotatable torsion by consensus in PG corresponds to a flexible torsion in VPG, whereas a locked torsion by consensus in PG corresponds to a rigid torsion in VPG), the AM equals 0. When the VPG fails to match the majority of PG designations, the AM varies towards 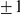 (−1  =  flexible and +1  =  rigid). The variance from 0 indicates the proportion of disagreement. To calculate the AM index for the *n-th* torsion, we implement the following algorithm defined as:
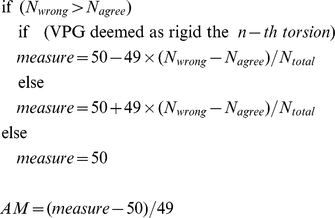
where 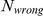 is the count of times that PG disagreed with VPG, 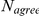 is the count of times that PG matched the VPG, and 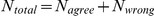 is the total number of realizations for the PG. For instance, if a particular torsion has a value of 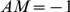, it indicates that the VPG assesses the torsion to be rigid, whereas the PG indicates the opposite (flexible) in all of the realizations. When 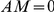, this is considered perfect agreement, and when 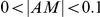, there is disagreement between the consensus PG vote and the VPG prediction, but the minority and majority votes from PG are very close, where the difference is comparable to the intrinsic sampling error bars.

### Rigidity Profiles

To complement the analysis above, we also graphically compare two additional descriptions of network rigidity that resemble contact maps. The *Rigid Cluster Map* (RCM) is a 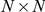 symmetric matrix that identifies co-rigid 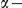
carbon pairs within protein structure. By definition the main diagonal is rigid (an 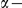
carbon is rigid with respect to itself). When constructing the RCM matrix, if a pair of 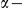
carbons belong to the same rigid cluster a value of 1 is assigned to the intersection of both vertices (specifying the row/column of the matrix), else 0 is given. For one run of the PG, the RCM is a binary plot simply highlighting co-rigid residue pairs. The 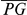 RCM plots are based on a majority rule across the ensemble. That is, if 50% or more of the realizations is rigid a 1 is assigned, otherwise 0 is assigned. For the VPG, there is only one run, and the output will be 1 or 0. Since the RCM is symmetric, the lower triangle shows the VPG, while the upper triangle shows the 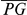 results.

Further, we also employ *Mechanical Coupling Maps* (MCM) to characterize how flexibility propagates throughout structure. The MCM quantifies the degree of flexibility of each 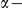
carbon in a protein relative to a reference 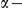
carbon, which serves as a rigid body anchor to eliminate the trivial rigid body translations and rotations. To calculate the MCM, the maximum number of excess DOF shared between the 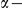
carbon of interest and the reference 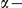
carbon must be determined. Operationally, this is accomplished by first fixing the trivial six DOF on the reference 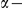
carbon, and then launch a pebble search on the other 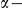
carbon to gather the maximum number of internal DOF. Note that the result does not depend on which 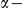
carbon is selected as a reference, as the result depends only on the 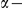
carbon pairs. For normalization purposes, the number of internal DOF found is divided by six (being the maximum number of DOF that an 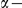
carbon can have). This information is presented using a color code scheme in the MCM that ranges from 0 to 1. Since the number of DOF that can be found in the VPG can be fractional (not binary like the RCM), the proper comparison to 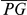 requires the MCM values from the PG runs to be averaged across the ensemble. The MCM thus provides a more nuanced view of network rigidity than the RCM. Because the MCM is also symmetric, again the lower triangle shows the VPG, while the upper triangle shows the 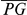 results.

## Results and Discussion

### Quantifying Rigid Cluster Similarity

Characterizing the rigid clusters offers a unique view in terms of the role that chemical interactions play within proteins. In prior work, we have used rigid cluster decomposition of protein structure to provide a statistically significant description of thermodynamic coupling within double mutant cycles [43]. Moreover, there have been many investigations characterizing the loss of rigidity that occurs upon protein unfolding using a H-bond dilution model [11], [34], [44]–[48]. Finally, PG characterizations of rigidity have been used to explain the increased stability of thermophilic proteins [49], RNA function [50], the effects of ligand binding [5] and the identification of critical interactions [51]. In these works, an energy cutoff is used to identify which H-bonds are present. As the energy cutoff is lowered, less H-bonds are included in the structure, thus increasing flexibility. Equivalently, as the energy cutoff is raised, more H-bonds are included in the structure, thus increasing rigidity.

In an analogous way, we vary 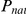 from 0 to 1 in order to control the number of H-bonds present in the network. One technical difference here, is we treat all H-bonds as equivalent, and ignore their energies altogether. In the above mentioned previous works, H-bond energy was used to characterize the strength of a H-bond so that the weakest H-bonds can be removed before the strongest H-bonds to study protein unfolding. The reason for *intentionally* treating all H-bonds as equivalent in this this work is because we are interested in testing how good the VPG can represent 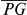 over the ensemble. If some H-bonds are almost always present (lowest energy H-bonds) while some H-bonds are almost always broken (high energy H-bonds), the VPG results will be closer to the 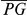 results because less fluctuations will occur in the H-bond network, meaning the comparisons herein correspond to the worst-case scenario. We have tested and verified this dependence on the fluctuations present in the H-bond network, and we will publish a more physically realistic H-bond dilution protocol elsewhere that models protein unfolding. However, the interest in this report is to show that the VPG provides an excellent approach to characterize protein flexibility/rigidity properties even in the extreme case of uniform H-bond strength.

Under this H-bond dilution strategy, the capacity defined by 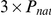 is the number of DOF that will be removed from the VPG when an H-bond is independent (recall each H-bond is described by three distance constraints). For the PG counter part, a random number between zero and one is assigned to each possible H-bond in each PG realization to determine if it is present or not (H-bond is present if 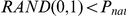). Note that empirically we find that for a given 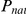, an ensemble of 200 realizations is typically good enough to make robust predictions across our protein dataset. Since we are using 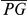 to define the exact answer to compare against the VPG, we run the PG 1000 times to reduce statistical error bars by 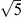 relative to what is found in actual applications.

**Figure 3:**
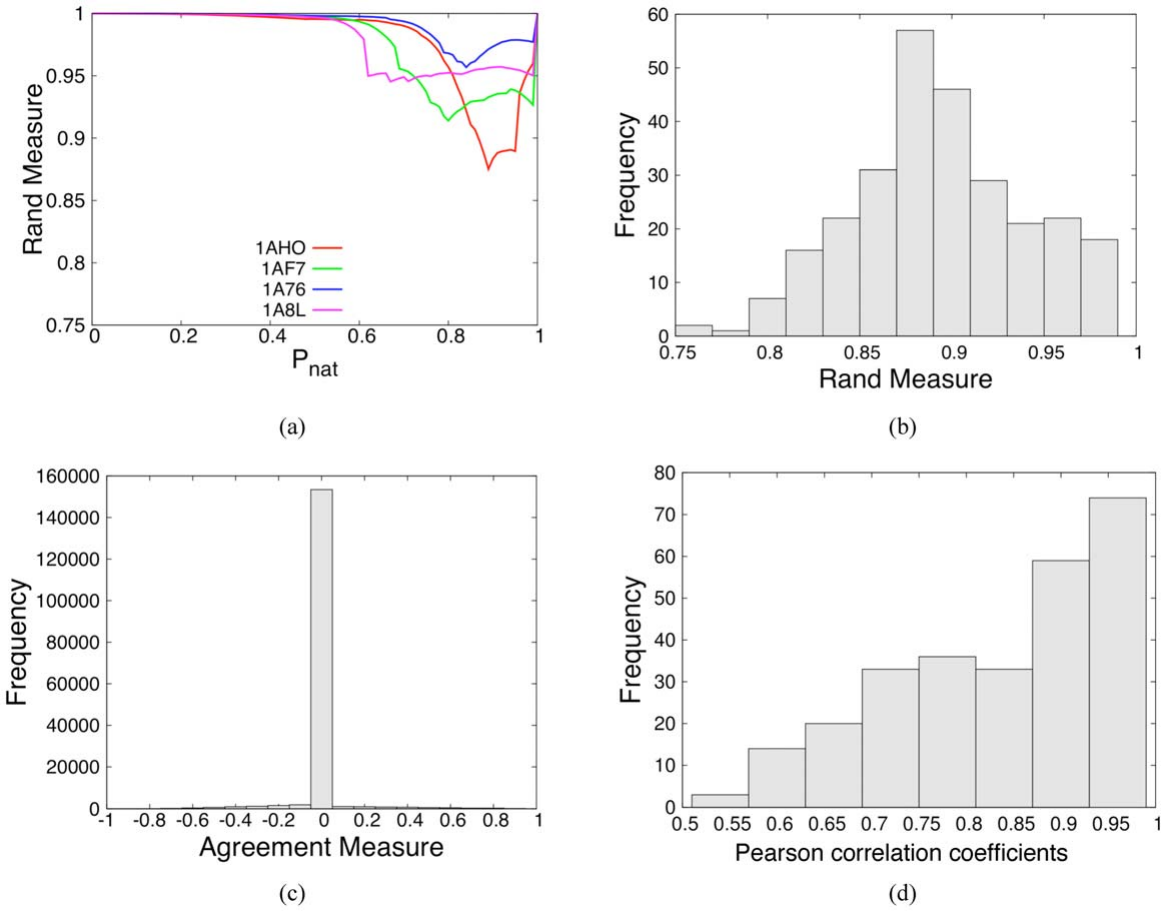
Quantifying 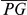 and VPG similarity. (a) The Rand Measure (RM) is plotted versus 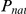 for four exemplar proteins that span a range of sizes (from 64 to 315 residues) and topological architectures. All proteins across the full dataset have the same characteristic shape where the minima in RM is related to the protein structure's rigidity transition. The 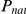 value corresponding to the worst RM is defined as 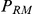. (b) Histogram detailing 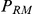 values for each protein within our dataset. Encouragingly, an overwhelming majority of cases have RMs greater than 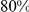. (c) Histogram detailing the agreement measure for each backbone torsion within our dataset at each protein's respective 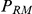 value. (d) Histogram detailing the Pearson correlation coefficient comparing the 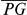 and VPG mechanical coupling maps across the dataset at each protein's respective 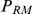 value.

As discussed above, the Rand Measure [42] (RM) compares the rigid cluster decompositions from the VPG and 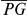. Fig. 3a presents the RM calculation across the range 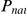 values for four exemplar protein structures. The four example proteins span a range of sizes (from 64 to 315 residues) and topological architectures. Specifically, they are the chemotaxis receptor methyltransferase CheR structure (pdbid  =  1AF7) [52], the FLAP endonuclease from *M. jannaschii* (pdbid  =  1A76) [53], a small scorpion protein toxin (pdbid  =  1AHO) [54] and the disulfide oxidoreductase from *P. furiosus* (pdbid  =  1A8L) [55]. In each, there is a region where the RM decreases sharply, which corresponds to the worst-case situation when the fluctuations are maximized. For most networks the point of maximum fluctuation identifies the rigidity threshold, representing the transition from flexible to rigid. A similar pattern was detected across our entire dataset, which appears at values as low as 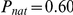 and ends at values as high as 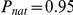. To calculate RM at each 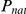, each one of the 1000 PG realizations is compared to the single VPG description. The 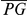 RM value is simply the average of the RM 1000 realizations. The 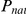 at the minimum in RM, designated 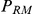, identifies the worst-case scenario.

**Figure 4:**
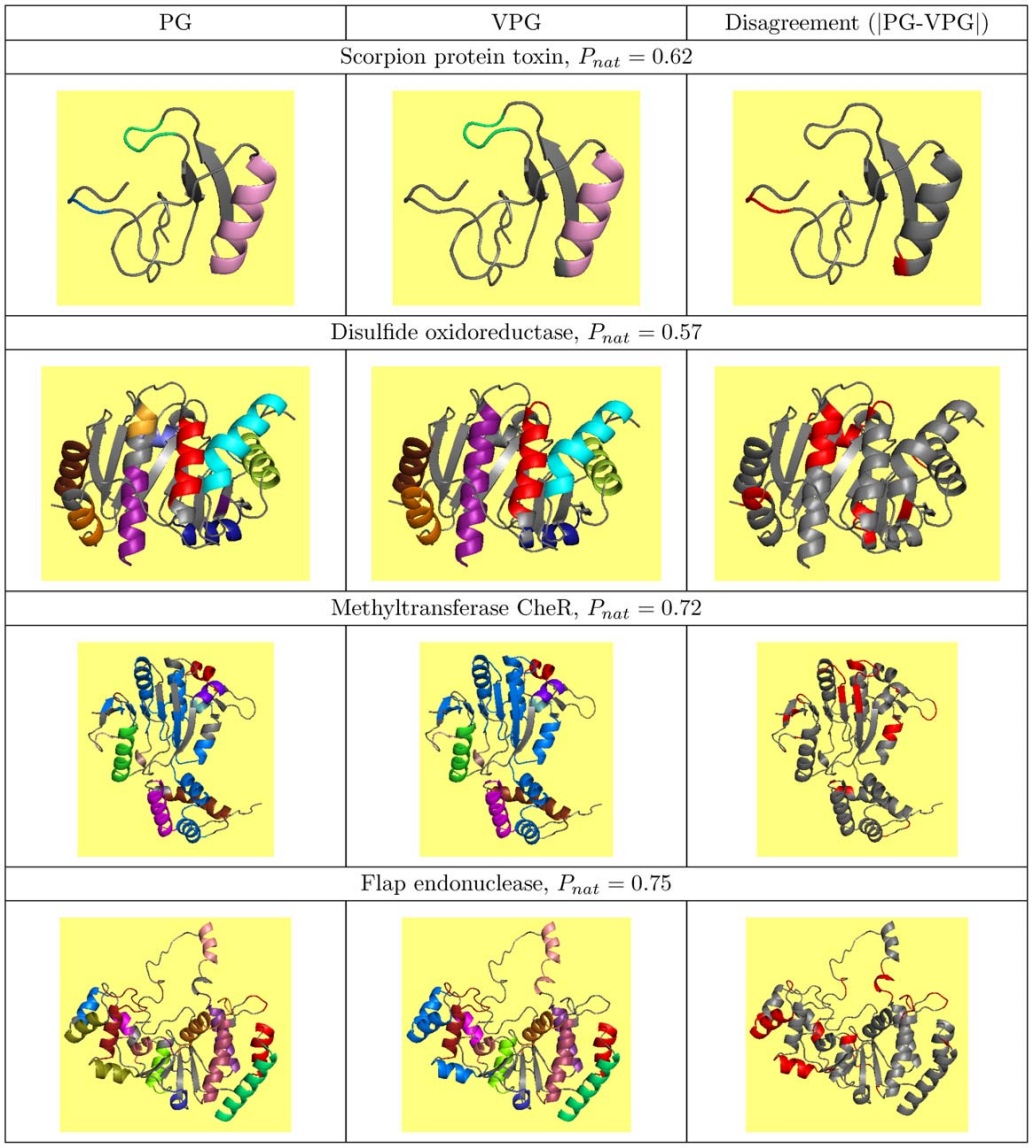
Rigid Cluster visualizations for four example proteins. The first column highlights the rigid clusters identified within the PG realization that corresponds to the median RM value, designated 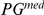. The middle column corresponds to the rigid clusters identified by the VPG. Finally, differences between the two algorithms are highlighted in the third column.

The high RM values indicate that the rigid cluster decomposition is very similar across the 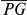 and VPG. To emphasize this point, we identify the PG realization that yields the median RM score across the entire ensemble, which is called 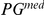. This rigid cluster decomposition for this point is plotted (using the same four proteins) in the first column of Fig. 4. Color differences indicate different rigid clusters, whereas grey indicates a flexible region. The middle column identifies the rigid clusters identified by the VPG. While the similarity is apparent by just qualitatively comparing the rigid cluster decompositions from each algorithm, the difference plots in the third column are the most compelling. Red coloring identifies regions that disagree in rigid cluster composition, whereas grey indicates agreement.

Expanding to our entire dataset, Fig. 3b plots a histogram of all RM scores at the respective 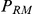 value for each protein. The worst-case RM scores are encouragingly large (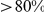) for an overwhelming majority of the proteins, thus indicating that the rigid clusters identified by the two algorithms are quite similar. Slight shifts to the considered 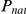 to just above and below 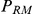 negligibly affects the histograms. Note that when 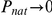 or 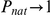 the fluctuations within the network are suppressed, meaning the algorithms become identical. Consequently, the mechanical descriptions converge. Fig. 3a typifies this result, where the two approaches produce identical results (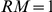) at small and large values of 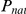.

### Over versus Under Prediction of Rigidity

**Figure 5:**
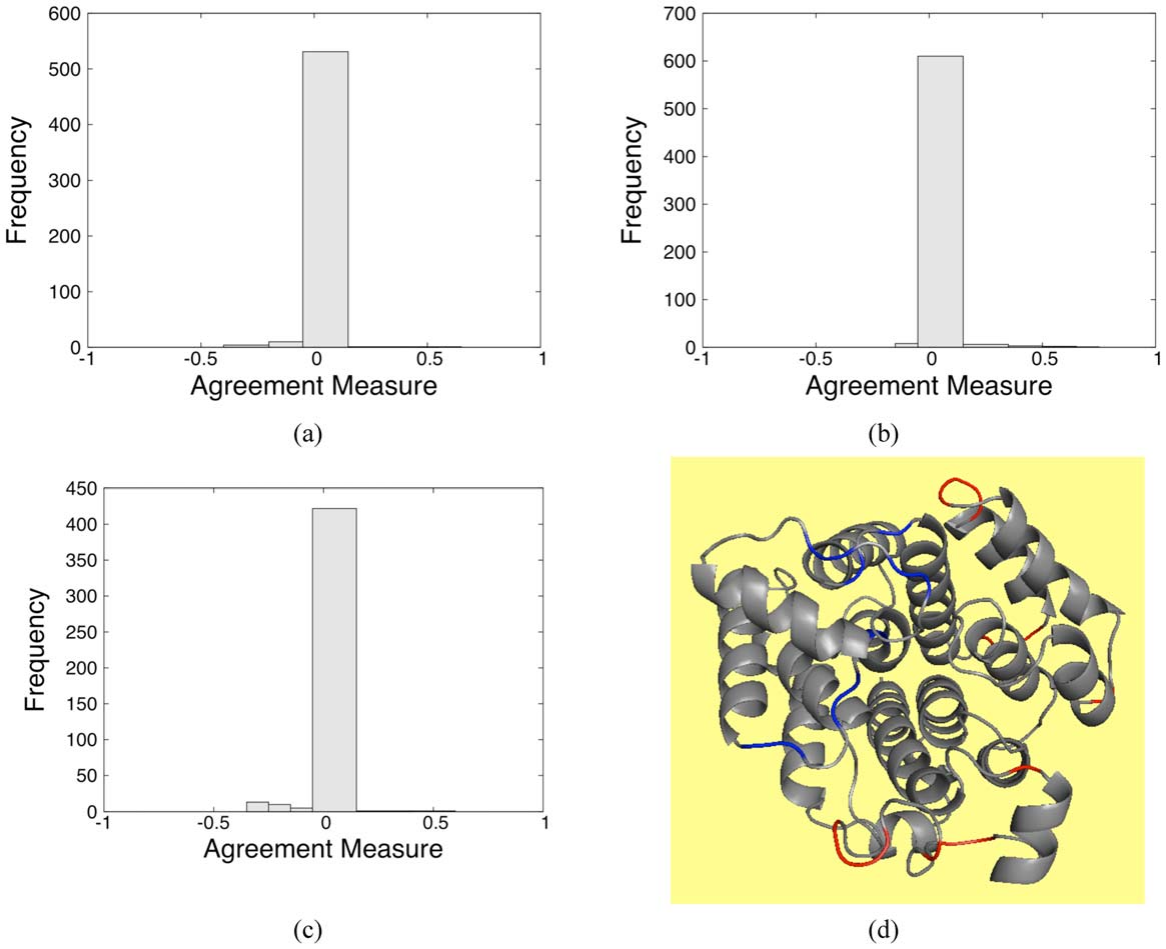
Agreement measure (AM) results. AM histograms for the (a) methyltransferase, (b) FLAP endonuclease and (c) disulfide oxidoreductase at their respective 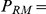 values. (d) Differences between the 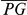 and VPG are mapped to the ribosylglycohydrolase structure from *M. jannaschii*, which is presented as a typical case. Red coloring indicates that the VPG overestimates rigidity relative to 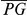, whereas blue indicates an underestimate. Across our dataset, as shown in this example, differences occur most frequently in loop regions.

The RM indicates that differences in the rigid cluster decomposition for PG and VPG occur, but the RM does not characterize where and how the differences take place. Therefore, it is important to determine if the VPG tends to systematically over- or under-estimate rigidity in the protein. To determine how often each type of error appears, we quantify similarity within the rigidity of all rotatable backbone (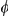 and 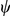) dihedral angles (torsions). The agreement measure (AM) described above is applied here for three specific proteins, and across the entire dataset. Fig. 5a, b and c present histograms of AM values for three of the proteins from above. In panel (a), it is shown that the VPG slightly overestimates the amount of rigidity within the methyltransferase CheR structure, whereas panel (b) indicates that it slightly underestimates the amount of rigidity within FLAP endonuclease. Panel (c) shows that VPG overestimates the amount of rigidity within the disulfide oxidoreductase. Fig. 3c presents a histogram of the entire dataset. Clearly, the overwhelming majority (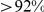) of torsions are in close agreement within statistical uncertainty of 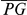. Strong disagreement (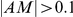) between both algorithms is minimal, especially considering the comparison is at the 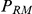 of each protein that defines the worst-case. Nonetheless, it is interesting to identify when discrepancies are most likely to occur. A survey of the differences reveals that they generally occur in loop regions and edges of secondary structures, as typified in Fig. 5d.

### Rigidity Profile Similarity

**Figure 6:**
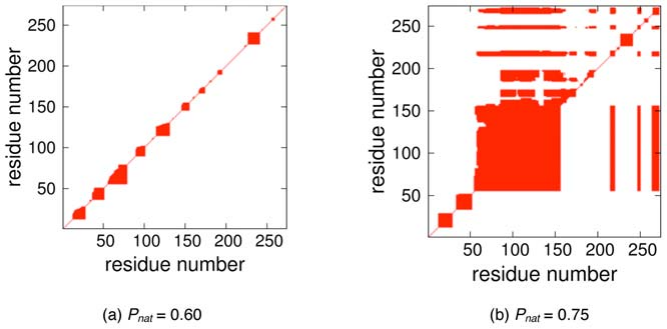
Rigid cluster maps (RCM) of chemotaxis receptor methyltransferase CheR structure is plotted at two different 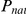 values. Red coloring identifies residue pairs that are co-rigid. 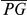 results are presented in the upper triangle, whereas the VPG is presented in the lower. At 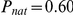, the protein is mostly flexible due to a lack of crosslinking H-bonds. However, the structure becomes increasingly rigid as H-bonds are added to the network. At 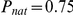, the VPG slightly under-predicts the extent of rigidity. For this protein 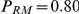.

We use Rigid Cluster Maps (RCM) to visually highlight pairwise mechanical couplings within structure, using red marks to highlight 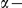
carbon pairs within the same rigid cluster, otherwise no mark is provided. For ease of comparison, the 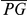 results are presented in the upper triangle, whereas the VPG results are presented in the lower triangle. By construction, the protein backbone corresponding to the RCM diagonal is always rigid in both variants. Using the methyltransferase structure from above as a typical case, the two panels in Fig. 6 correspond to two different values of 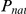, ranging from a completely flexible (unfolded) structure with few crosslinking H-bonds to a predominantly rigid structure with many crosslinking H-bonds. As one can see, the VPG and 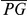 algorithms give very similar results.

**Figure 7:**
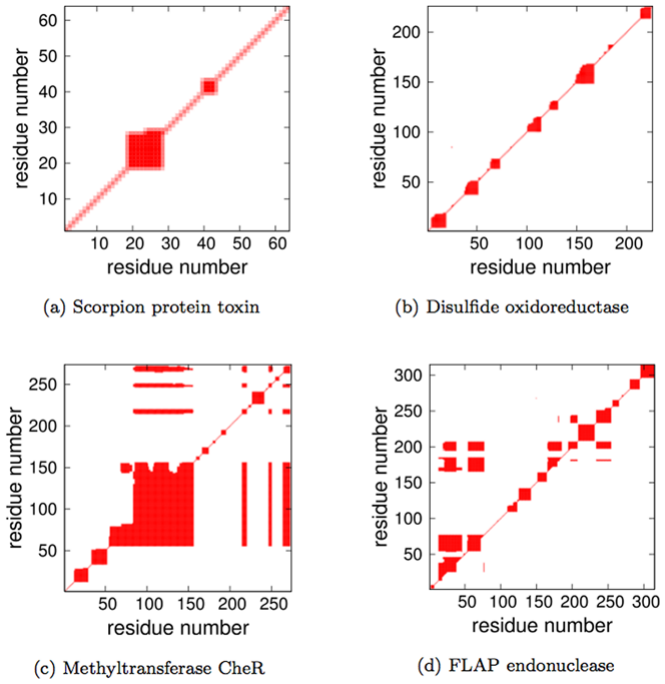
Rigid Cluster Maps (RCM) for four different example proteins near their respective 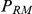 values. 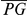
 results are presented in the upper triangle, whereas the VPG is presented in the lower. Note that the presented proteins are the same from Fig. 3a.

Going a step further, Fig. 7 presents the RCMs of our four example proteins near 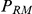, thus 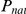 is corresponding to the critical region. The presented values are slightly shifted from exact 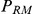 values to highlight interesting features. Note that the changes in 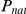 actually make the RCM plots appear more dissimilar. The large square region along the backbone corresponds to a rigid 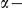
helix. A similar pattern is observed in the disulfide oxidoreductase, which also has few crosslinking interactions at this value of 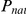. Conversely, the off-diagonal features are mostly conserved in the methyltransferase CheR structure, but the VPG slightly overestimates the extent of rigidity within the core region (residues 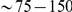). The FLAP endonuclease example provides the most interesting visual differences between the two approaches, where the VPG underestimates the 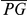 predictions. That is, the VPG fails to identify rigid clusters present within the 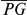. However, the differences are found to be much less severe on closer inspection regarding the number of available DOF. While there are no free pebbles within the 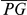 in these regions, the probabilistic VPG identifies a tiny nonzero fraction (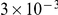). Clearly, this difference is negligible, but the binary RCM makes the difference appear much larger than it actually is.

**Figure 8:**
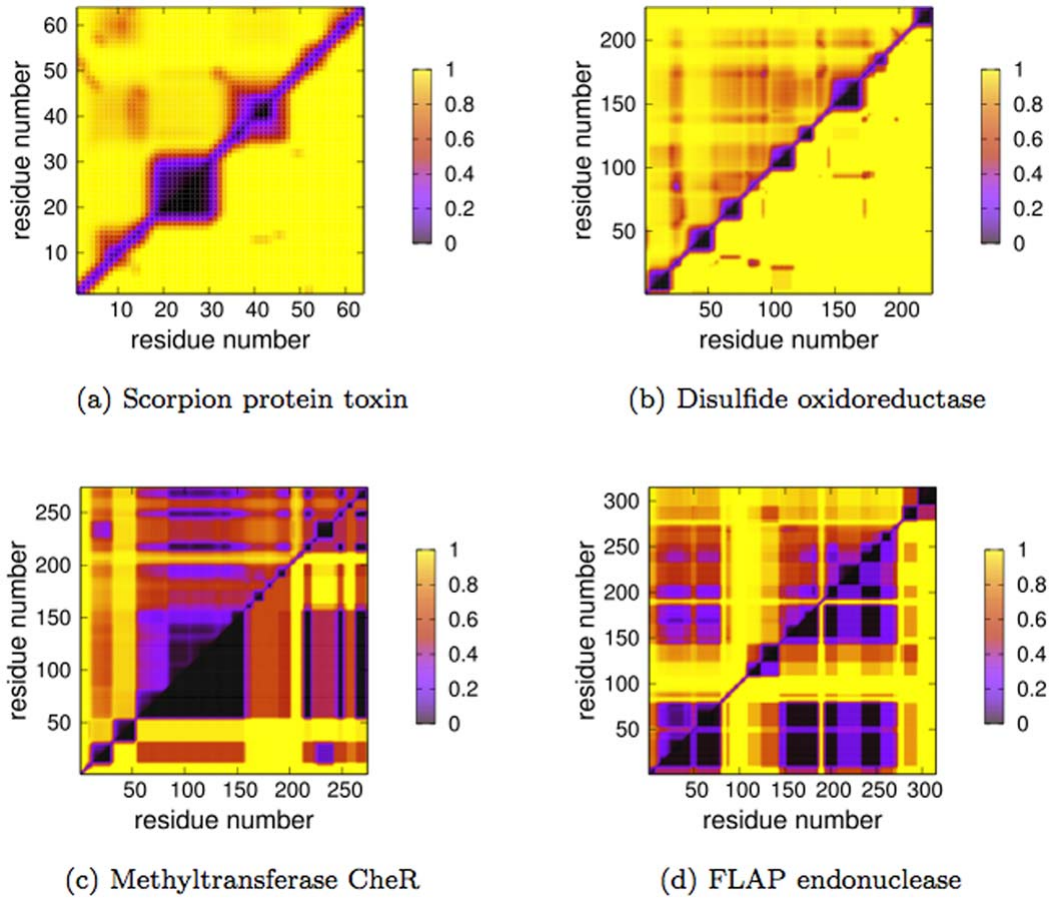
Mechanical Coupling Maps (MCM) provide a more nuanced description of co-rigidity. Specifically, the continuous scale provides a normalized description of how many free pebbles (DOF) are shared between each residue pair (0 = 0 pebbles, whereas 1 = 6 pebbles). Again, each MCM is plotted near their respective 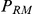 values for the same four proteins presented Figs. 3a and 7.

The Mechanical Coupling Maps (MCM) provide a more nuanced view of rigid cluster decomposition. Unlike the binary RCMs, MCMs are based on a continuous scale that identifies the fractional number of pebbles shared between each 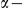
carbon pair. In this sense, the MCMs are similar to the cooperativity correlation plots calculated by our statistical mechanical DCM [18], [19], [22]–[25]. Fig. 8 compares the MCMs for the same four proteins in Fig. 7, using the same 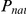 values. The rigidity over-prediction by the VPG in the methyltransferase example is again clear. However, there is appreciable co-rigidity within the residue pairs contained within the range of residues 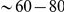 and residues 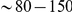, which was identified as flexible by the the RCM. Additionally, the MCMs reveal a more interesting set of similarities throughout the plots. In the same way, the similarity within FLAP endonuclease is also more pronounced, although the VPG again somewhat overestimates the extent of rigidity. Conversely, the MCMs actually show more dissimilarity within the two examples without any off-diagonal RCM components. In both, there is marginal co-rigidity identified by the 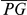 (the reddish shadowing) due to some rigidity fluctuations throughout the ensemble that is suppressed by the VPG.

**Figure 9:**
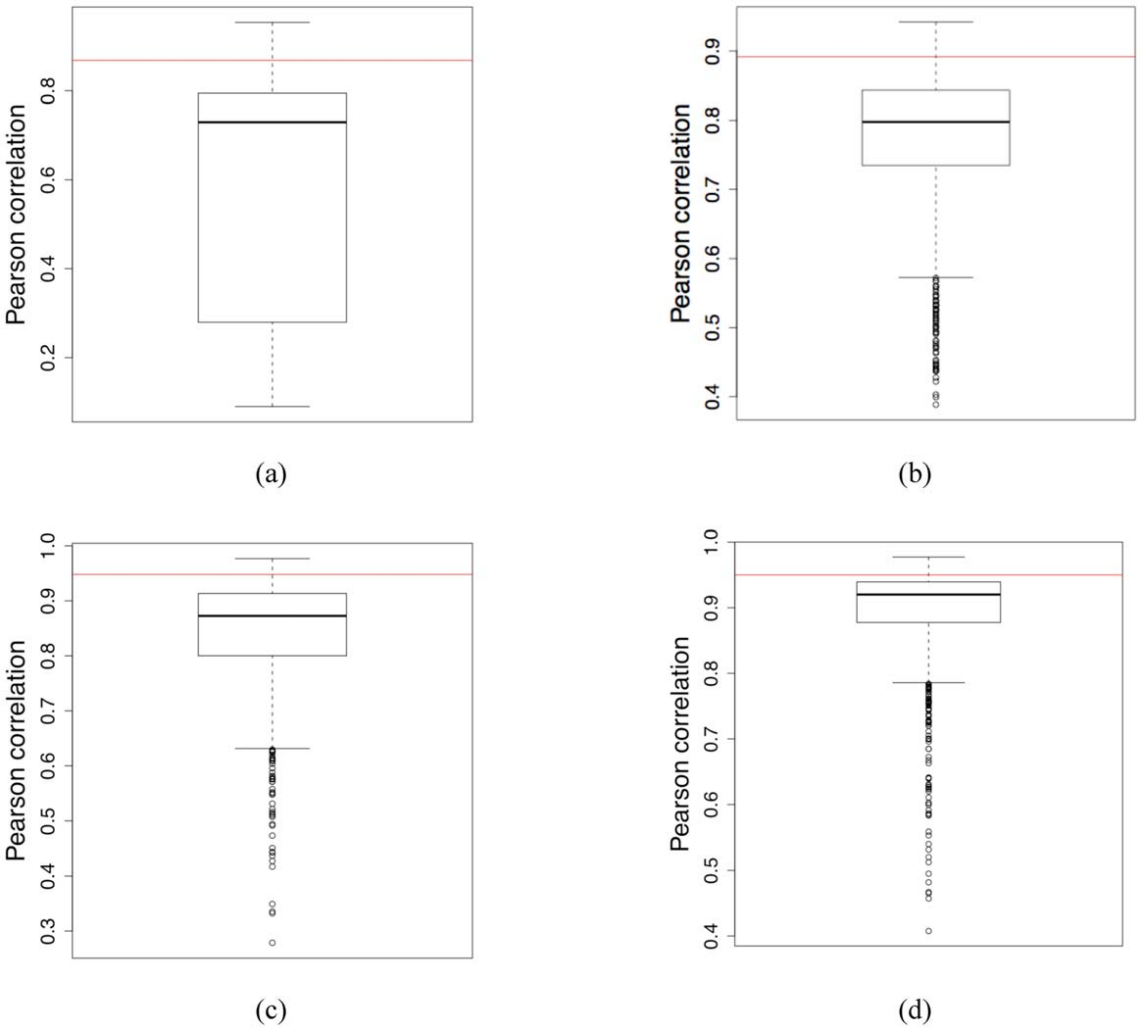
Boxplots describing the ensemble of Pearson correlations coefficients comparing each PG realization to the 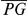 behavior. The red line represents the correlation between the 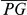 and the VPG. In all cases, the 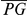 to VPG similarity is greater than the 75th percentile of the intrinsic fluctuations within the PG ensemble.

Expanding across the entire dataset, Fig. 3d provides a histogram of the Pearson correlation coefficient between the MCM matrices calculated by the 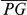 and the VPG. Clearly, the VPG is consistently a good estimator of the 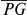 behavior. This point is strengthened by Fig. 9, which compares the Pearson correlation coefficients of the MCM of each unique PG realization to the 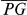 plot for the same four proteins considered above. That is, the boxplots describe the intrinsic variability across the PG ensemble. Within each boxplot, the horizontal grey line indicates the median RM value across the 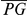 distribution, whereas the top and bottom of the box indicates the upper and lower quartiles. The whiskers describe the rest of the distribution, and the dots identify outliers (corresponding to the default settings of R). The red line corresponds to the similarity between the 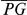 and VPG MCMs, which is encouragingly strong. In fact, the VPG similarity to the 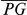 behavior is better than the third quartile in all cases. This result clearly indicates that VPG approximates 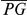 behavior better than the vast majority of the single PG realizations. These comparisons are calculated at the same value of 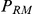 as above, meaning they again correspond to the critical region.

### The Rigidity Transition

**Figure 10:**
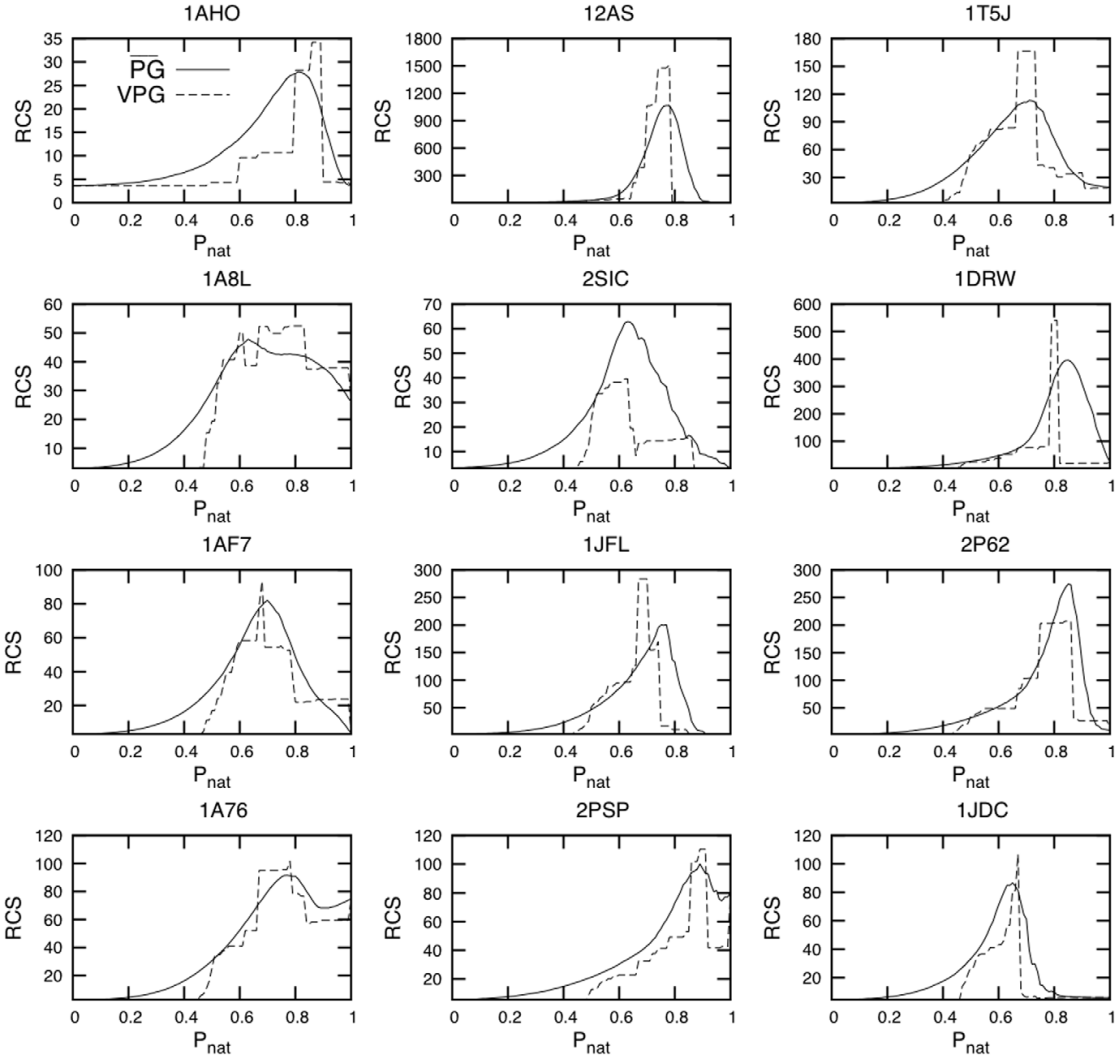
Rigid Cluster Susceptibility (RCS) is plotted versus 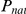 for 12 typical protein examples (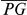  =  solid line and VPG  =  dashed line). Note that the proteins presented in the first column are the same from Fig. 3a.

**Figure 11:**
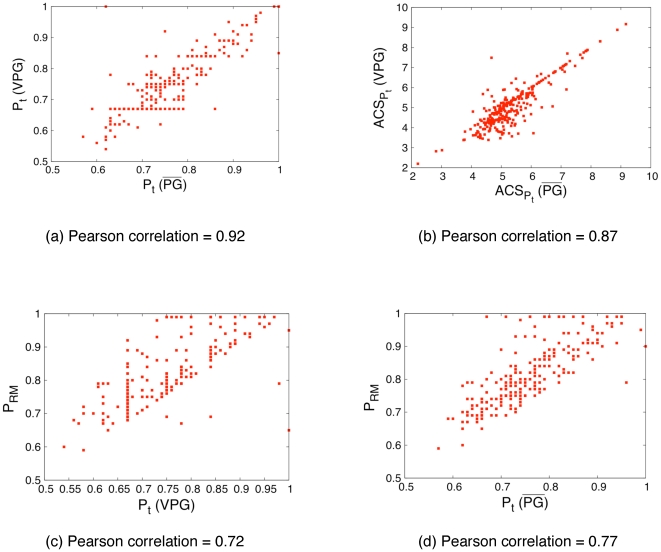
Rigidity transition effects. **(a) The rigidity transition (** 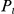 **) is compared across the** 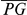 **and VPG algorithms.** (b) Similarly, the average cluster size (ACS) at their respective 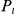 values are compared across the two algorithms. The value of 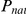 with the worst RM (called 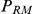) is compared to 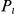 calculated using the (c) VPG and (d) 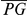. The linear relationships occur because the mean field approximation is maximally inaccurate in this range. Note, a few proteins do not have completed peaks in their rigid cluster susceptibility curves because the protein never crosses the rigidity transition, which have been excluded from panels (c) and (d).

Following earlier works [18], [19], [22], [25], [34], we define 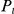 (for transition) as the peak in the rigid cluster susceptibility (RCS) curve, which is defined as the reduced second moment in rigid cluster size. That is, the peak in RCS identifies the point in which the rigid cluster sizes are maximally fluctuating, indicating a transition from a globally flexible to globally rigid network. Twelve examples (including the four proteins discussed above) of RCS curves using the 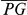 and VPG approaches are shown in Fig. 10, all of which are qualitatively similar. As shown by the scatter plot in Fig. 11a, the rigidity transitions identified by the 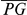 and VPG algorithms are highly correlated. In addition, the Average Cluster Size (ACS) at 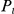 is also highly correlated across the two algorithms (Fig. 11b). Since 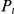 identifies the 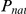 value with maximal variability within the rigid cluster sizes, it is expected to be related to the 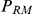 because the single VPG mean-field calculations suppresses fluctuations. This is indeed the case as indicated by the strong correlation between 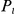 and 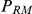 for both the 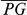 and VPG algorithms (Fig. 11c–d).

### Conclusions

In this report, we demonstrate that ensemble averaged 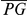 properties, which requires sampling, is approximated well by a single mean field calculation. That is, the probabilistic VPG accurately reproduces a number of ensemble-averaged network rigidity properties. The high values of the RM clearly indicate that the rigid cluster decompositions are very similar, especially for 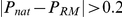. The AM and structural comparisons of the rigid clusters respectively provide quantitative support for this point. Comparisons of the RCM and MCM rigidity profiles between the 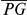 and VPG variants also indicate that the calculated rigidity properties are highly similar. In fact, the 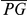 to VPG MCMs are much more similar than the intrinsic variability across the ensemble of PG snapshots. Finally, the mechanical transitions identified by the peak in the rigid cluster susceptibility curves are highly correlated across the two variants. Taken together, these results collectively demonstrate the utility and power of the virtual pebble game that deals with the probability of finding pebbles rather than the pebbles themselves.
